# Supplementary material for: Role and mechanism of NCAPD3 in promoting malignant behaviors in gastric cancer
Source: Front Pharmacol. 2024 Apr 22;15:1341039. doi: 10.3389/fphar.2024.1341039 (PMC11070777; doi:10.3389/fphar.2024.1341039)
Supplement: Supplementary file 11 [file DataSheet2.ZIP › GSEA/immunologic/my_analysis.Gsea.1599462003817/GSE45365_WT_VS_IFNAR_KO_CD11B_DC_UP.html]

Details for gene set GSE45365\_WT\_VS\_IFNAR\_KO\_CD11B\_DC\_UP[GSEA]

|  || Dataset | filtered\_dataset.sample\_info.cls#WT\_versus\_NCAPD3\_MUT |
| Phenotype | sample\_info.cls#WT\_versus\_NCAPD3\_MUT |
| Upregulated in class | WT |
| GeneSet | GSE45365\_WT\_VS\_IFNAR\_KO\_CD11B\_DC\_UP |
| Enrichment Score (ES) | 0.3959111 |
| Normalized Enrichment Score (NES) | 1.7749511 |
| Nominal p-value | 0.008250825 |
| FDR q-value | 0.9829252 |
| FWER p-Value | 1.0 |
Table: GSEA Results Summary

  

Fig 1: Enrichment plot: GSE45365\_WT\_VS\_IFNAR\_KO\_CD11B\_DC\_UP      
 Profile of the Running ES Score & Positions of GeneSet Members on the Rank Ordered List

  

| SYMBOL | TITLE | RANK IN GENE LIST | RANK METRIC SCORE | RUNNING ES | CORE ENRICHMENT || 1 | 63926 | ANKEF1 | 4 | 1.180 | 0.0783 | Yes |
| 2 | 3954 | LETM1 | 47 | 0.924 | 0.1117 | Yes |
| 3 | 117145 | THEM4 | 65 | 0.860 | 0.1586 | Yes |
| 4 | 23338 | JADE2 | 71 | 0.855 | 0.2138 | Yes |
| 5 | 10207 | INADL | 73 | 0.851 | 0.2716 | Yes |
| 6 | 55687 | TRMU | 128 | 0.774 | 0.2860 | Yes |
| 7 | 92935 | MARS2 | 143 | 0.741 | 0.3269 | Yes |
| 8 | 84976 | DISP1 | 221 | 0.659 | 0.3168 | Yes |
| 9 | 91574 | C12orf65 | 238 | 0.646 | 0.3497 | Yes |
| 10 | 152485 | ZNF827 | 268 | 0.618 | 0.3714 | Yes |
| 11 | 54758 | KLHDC4 | 292 | 0.598 | 0.3959 | Yes |
| 12 | 27340 | UTP20 | 455 | 0.501 | 0.3137 | No |
| 13 | 6835 | SURF2 | 582 | 0.434 | 0.2529 | No |
| 14 | 219743 | TYSND1 | 607 | 0.419 | 0.2644 | No |
| 15 | 58509 | CACTIN | 748 | 0.351 | 0.1878 | No |
| 16 | 54532 | USP53 | 898 | -0.322 | 0.1026 | No |
| 17 | 4209 | MEF2D | 910 | -0.337 | 0.1179 | No |
| 18 | 1978 | EIF4EBP1 | 938 | -0.362 | 0.1234 | No |
| 19 | 27250 | PDCD4 | 1170 | -0.537 | -0.0060 | No |
| 20 | 57494 | RIMKLB | 1233 | -0.610 | -0.0087 | No |
| 21 | 8651 | SOCS1 | 1272 | -0.661 | 0.0095 | No |
| 22 | 51274 | KLF3 | 1406 | -1.296 | 0.0029 | No |
Table: GSEA details [plain text format]

  

Fig 2: GSE45365\_WT\_VS\_IFNAR\_KO\_CD11B\_DC\_UP      
 Blue-Pink O' Gram in the Space of the Analyzed GeneSet

  

Fig 3: GSE45365\_WT\_VS\_IFNAR\_KO\_CD11B\_DC\_UP: Random ES distribution      
 Gene set null distribution of ES for **GSE45365\_WT\_VS\_IFNAR\_KO\_CD11B\_DC\_UP**
